# Complex responses to movement-based disease control: when livestock trading helps

**DOI:** 10.1098/rsif.2016.0531

**Published:** 2017-01

**Authors:** Jamie C. Prentice, Glenn Marion, Michael R. Hutchings, Tom N. McNeilly, Louise Matthews

**Affiliations:** 1 Institute of Biodiversity, Animal Health and Comparative Medicine, College of Medical, Veterinary and Life Sciences, Glasgow G61 1QH, UK; 2 Boyd Orr Centre for Population and Ecosystem Health, Institute of Biodiversity, Animal Health and Comparative Medicine, College of Medical, Veterinary and Life Sciences, University of Glasgow, Glasgow G12 8QQ, UK; 3 Biomathematics and Statistics Scotland, Edinburgh EH9 3FD, UK; 4 Disease Systems, SRUC, Edinburgh EH9 3JG, UK; 5 Moredun Research Institute, Penicuik EH26 0PZ, UK

**Keywords:** basic reproduction ratio, *Escherichia coli* O157, bovine viral diarrhoea virus, *Mycobacterium avium* ssp. *paratuberculosis*, heterogeneity, supershedder

## Abstract

Livestock disease controls are often linked to movements between farms, for example, via quarantine and pre- or post-movement testing. Designing effective controls, therefore, benefits from accurate assessment of herd-to-herd transmission. Household models of human infections make use of R_*_, the number of groups infected by an initial infected group, which is a metapopulation level analogue of the basic reproduction number *R*_0_ that provides a better characterization of disease spread in a metapopulation. However, existing approaches to calculate R_*_ do not account for individual movements between locations which means we lack suitable tools for livestock systems. We address this gap using next-generation matrix approaches to capture movements explicitly and introduce novel tools to calculate R_*_ in any populations coupled by individual movements. We show that depletion of infectives in the source group, which hastens its recovery, is a phenomenon with important implications for design and efficacy of movement-based controls. Underpinning our results is the observation that R_*_ peaks at intermediate livestock movement rates. Consequently, under movement-based controls, infection could be controlled at high movement rates but persist at intermediate rates. Thus, once control schemes are present in a livestock system, a reduction in movements can counterintuitively lead to increased disease prevalence. We illustrate our results using four important livestock diseases (bovine viral diarrhoea, bovine herpes virus, Johne's disease and *Escherichia coli* O157) that each persist across different movement rate ranges with the consequence that a change in livestock movements could help control one disease, but exacerbate another.

## 1. Background

Livestock diseases have an important impact not only on the economy and animal welfare [1,2], and can also pose a zoonotic risk to humans [3–5]. Many are introduced into herds via movements of infected animals, e.g. bovine tuberculosis (bTB), brucellosis, bovine viral diarrhoea (BVD), scrapie, foot-and-mouth disease (FMD) and Johne's disease [5–11]. Livestock disease control is therefore often implemented at the point of between-farm movement [12,13]. Controls that target infected animals moving between farms, include vaccination, quarantine, restricting movement for farms found to have infected animals, or even international movement restrictions [14]. This leads us to the question of how hard a disease is to control when control is directed at herd-to-herd spread and how disease control effort depends on the rate of livestock movement.

The usual metric for assessing the required degree of control is the basic reproduction ratio, *R*_0_ [15–17]. *R*_0_ is the number of secondary infectives following introduction of a single typical primary infected individual into an entirely susceptible population. If *R*_0_ > 1, then a disease can invade, whereas if *R*_0_ ≤ 1 it cannot. The aim of disease intervention is often described in terms of reducing effective *R*_0_ to below 1. For example, if a proportion *q* of the population is vaccinated, then the effective *R*_0_ is *R* = (1 − *q*)*R*_0_, giving the critical coverage to prevent disease spread of *q*_c_ = 1 − 1/*R*_0_ [15,18].

However, *R*_0_ is an individual-based rather than a group-based metric, and a system with high *R*_0_ could have high within-group (i.e. within farm) transmission, but only low between-group (between farm) transmission [19]; *R*_0_ can therefore be poor at describing transmission within metapopulations [20], such as the risk of disease in one farm spreading to others, e.g. via livestock movements.

There have been several approaches to address this deficiency. Early patch-based models proved analytically tractable, but only considered the infected status of a patch as a whole, and assumed that the timescale of reaching a quasi-stationary state was short relative to movement dynamics [21–23]. This sort of simple model has sometimes failed to predict more complex and unintuitive disease dynamics [24]. Household models examine disease persistence within a metapopulation of a large number of small groups (e.g. households), and typically assume that disease spreads between groups that share individuals (e.g. children mix with other children at school, the adults mix with other adults at work and both return to the household), or that proximity is sufficient (e.g. transmission between patches of plant populations) [25–27]. However, household models neglect more long-lived movements such as those from livestock moving between farms, or wildlife dispersing from their natal range [25,28,29], and in doing so ignore the depletion of infectives from the primary group.

In the context of household models, Ball & Neal [30–32] introduce R_*_, a group-level analogue of *R*_0_, describing the number of secondary infected groups generated by a primary infected group. As with *R*_0_, R_*_ = 1 provides a threshold for disease spread within the metapopulation. Similarly, 1 − 1/R_*_ provides the degree of disease intervention necessary to prevent disease spread. In situations where disease control is at the group-to-group level, R_*_ provides a convenient alternative to *R*_0_ for predicting levels of disease control required, especially in highly heterogeneous metapopulations.

Here, we derive R_*_ using the next-generation matrix (NGM) technique [33,34], for a generic metapopulation model with disease spread via explicit animal movements that *does* account for the depletion of susceptibles from the primary group. In the simplest case, R_*_ takes an intuitive form in terms of the movement rate, the within-herd prevalence and the within-herd persistent time. We demonstrate that R_*_ peaks at intermediate movement rates, revealing ranges of movement rates where disease intervention will be most difficult.

We illustrate our findings for four important livestock infections—bovine herpes virus (BHV), bovine viral diarrhoea virus (BVDV), *Mycobacterium avium* ssp *paratuberculosis* (paraTB) and *Escherichia coli* O157 (*E. coli* O157)—showing that a reduction in movement rates could counterintuitively result in an increase in disease prevalence, and moreover, that control to reduce one disease could exacerbate another.

### 1.1. The next-generation matrix approach

In a recent helpful overview of NGM approaches, Diekmann *et al*. cover their use over a wide range of single population disease models [34,35], but do not consider metapopulation models. The NGM provides a natural basis for the calculation of *R*_0_. In brief, the approach is to obtain a matrix ***K***, where the entries *K_ij_* represent the expected number of new cases with state-at-infection *i*, arising from one individual with state-at-infection *j*. *R*_0_ is the dominant eigenvalue of this matrix.

To illustrate the technique, consider a single population with SEIR disease dynamics:
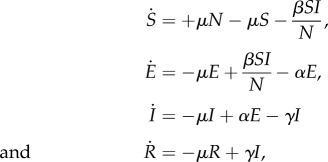
where individuals are born into susceptible state *S*, following infection they enter exposed state *E* and incubate the disease, then progress to the infectious state *I*, and finally recover to the immune state *R*. *N* is the population size, *μ* is the *per capita* mortality rate, here set equal to the birth rate, *β* is the disease transmission coefficient, 1/*α* is the average incubation period and *γ* is the *per capita* recovery rate. The state space is the vector 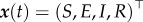.

To obtain ***K***, first linearize around the disease-free equilibrium, 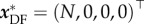, giving for small *E* and *I* the linearized infectious subsystem
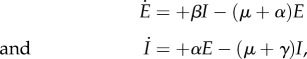
where only the production of new infectives and changes in the state of existing infectives are captured. The linearized subsystem is the form 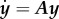, where 
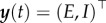
 and
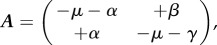
is the Jacobian matrix.

Now, decompose ***A*** into the sum of two matrices 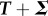, where
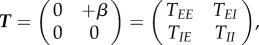
is the matrix of *transmissions*, where *T_EI_* represents the rate at which newly infected individuals in state *E* are created by infectious individuals *I*, and
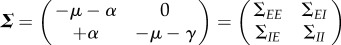
is the matrix of *transitions*, where, for example, 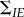 is the rate at which individuals move into state *I* from state *E*. Negative entries represent a net flow out of the state in question; hence, 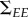 shows the rate at which individuals that start in *E* leave this class, without returning. The matrix
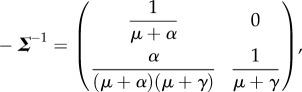
is interpreted biologically as the matrix of *sojourn times* [34]. Thus, the entries of the first column of matrix 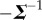 are the expected time spent in states *E* and *I* conditional on starting in state *E* (likewise entries of the second column are the expected times conditional on starting in state *I*).

The NGM with large domain, ***K***_L_, is given by the matrix product of transmission rate and residence time, that is 
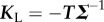
 [33], and so
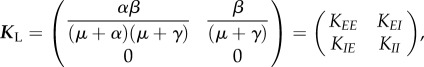
where, for example, *K_EI_* is the number of infections of type *E* generated by an index case in the *I* class. *R*_0_ is then the dominant eigenvalue of ***K***_L_
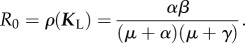


By including only the rows and columns of ***K***_L_ related to categories of state-at-infection (i.e. exposed *E*, but not infectious *I*), ***K***_L_ can be reduced to the NGM matrix ***K***
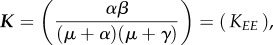
which is smaller and mathematically easier to work with, and has a biological interpretation convenient for direct construction using epidemiological principles [34]. The dominant eigenvalue is the same for both ***K***_L_ and ***K***, and either may be used to calculate *R*_0_.

In the scalar case above, *R*_0_ = ***K***_EE_, which, on taking the limits 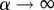 and 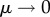, reduces to the familiar 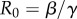 for the SIR model. This calculation for the SIR model also follows by identifying transmission ***T*** = *β* and the transition rate 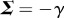, whence the time spent infectious is 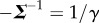, and the expected number of secondary infections from an index case in an otherwise susceptible population is 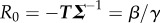. The NGM approach thus rigorously extends such arguments to more complex settings.

## 2. Next-generation matrix approach for homogeneous metapopulation dynamics with one disease category

We now apply the NGM approach to disease spread among a metapopulation of livestock herds, first illustrating the approach for a disease system with one disease category, and then showing how this may be naturally extended to more complex diseases. We show that R_*_ may be given by the intuitive formula
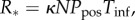
with *per capita* movement rate *κ*, herd size *N*, herd expected infectious lifetime *T*_inf_ and average prevalence of infectives during the infectious lifetime *P*_pos_. This is conceptually similar to the SIR model formula 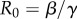 if one considers substituting the rate at which new infectious individuals are formed, *β*, with the average rate at which infectives leave herds *κNP*_pos_, and substituting in the expected infectious period, 1/*γ*, with the expected time disease persists in the herd, *T*_inf_.

### 2.1. Derivation of R_*_

Consider an SIS disease dynamic in a metapopulation of herds each containing *N* individuals. In the absence of infection, individuals die and are replaced with susceptibles at *per capita* rate *μ*. We assume frequency-dependent disease transmission with transmission rate *β*, recovery at *per capita* rate *γ* and a *per capita* movement rate between herds of *κ*.

For analytic tractability, we maintain constant herd size by assuming that the birth and death processes are coupled. Thus, the status of each herd may be defined by just the number of infectives, *i*, because the number of susceptibles is *s* = *N* − *i*. We consider a homogeneous metapopulation where we assume undirected movement between herds, and that movements are equally likely between any herds.

We choose to represent the metapopulation dynamics using the master equation approach (also known as the Chapman–Kolmogorov forward equation, see [36] for a detailed explanation) that allows us to capture the probability of a herd being in a state with *i* infectives. We begin by considering the respective rates *l*(*i*) and *g*(*i*) at which infectives are lost and gained. We assume when an animal leaves a herd it is replaced with a susceptible or infected individual in proportion to their prevalence in the metapopulation. Consequently, *l*(*i*) and *g*(*i*) depend on *P*_S_ and *P*_I_, the mean prevalence of susceptibles and infectives in the metapopulation, i.e.
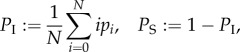
where *p_i_* is the probability that a herd contains *i* infectives.

In a herd with *i* infectives, the net loss of infectives owing to movements is 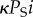 (because a proportion *P*_S_ of replacements are susceptible); therefore, the net loss of infectives via mortality, recovery and movement is
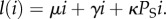


Similarly, the net gain of infectives owing to movements is equal to the net loss of susceptibles, which is given by 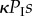. Therefore, the net gain of infectives via disease transmission and movement is
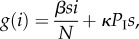
where *s* = *N* − *i*.

We may now write down the master equation governing the probability *p_i_*(*t*) of a herd containing *i* infectives at time *t*:
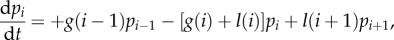
for *i* = 1,2, … ,*N* and subject to
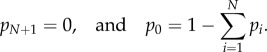


Here, 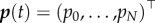 is a vector of length *N* + 1. Removing the disease-free state *i* = 0, gives ***q***(t), a vector of length *N* describing the probability of *i* infectives in the infectious subsystem.

To determine R_*_, we first linearize around the disease-free state 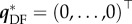. For *q_i_* close to the disease-free state for *i* = 1, … ,*N*, we obtain
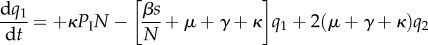
and
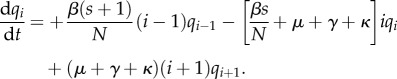


This can be written in matrix form
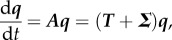
where ***A*** is the Jacobian matrix, and is decomposed into 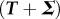, where ***T*** is the matrix of transmissions, and 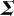 is the matrix of transitions. Here
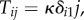
where *δ_ij_* = 1 if *i* = *j* and 0 otherwise, i.e.
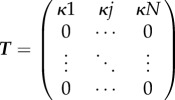
and
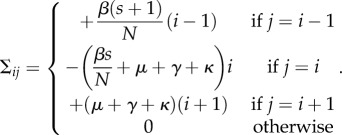


For the metapopulation model, the interpretation of ***T*** and 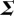 differs from the single population model as follows. In a single herd model, ***T*** describes the production of new infections via within-herd transmission [34]; however, in the metapopulation model, it represents the production of new infected *herds* via movement of infected individuals from an infected herd to a susceptible one. In a single herd model, 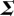 represents transitions between different disease states; in the metapopulation model, it represents the transitions between different states (in this case, different numbers of infectives) of an infected herd via within-herd transmissions, recoveries or mortalities, and movement of infectives to already infected herds.

As above, the matrix 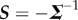 is the matrix of *sojourn times*, where the entry *S_ij_* is the expected time that a herd currently observed in state *j* will thereafter spend in state *i*. Because infected herds are assumed to begin with a single infective (i.e. *i* = 1), the total expected infectious period, *T*_inf_, is the sum of the times spent in each state, i.e. the sum of the entries in *column* 1, gives
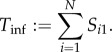


As ***T*** is zero everywhere other than the first row, the NGM of large domain 
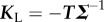
 is also zero everywhere except the first row (in this case, the dominant eigenvalue of ***K***_L_ is equal to the first entry of ***K***_L_). The only state-at-infection is *I*_1_, and so ***K*** = [***K***_L_]_11_. Thus, R_*_ is given by
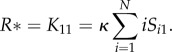


The expected proportion of time spent by a herd in state *i*, having started in state 1, is given by *S_i_*_1_/*T*_inf_. Using this, we now define the expected prevalence in an infected herd, *P*_pos_, by
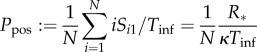


Rearranging, we obtain2.1
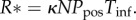


Therefore, R_*_, the expected number of secondary infected herds, is (intuitively) given by the product of the expected rate at which infectives leaving a herd (*κNP*_pos_), and the duration of the infection in a herd (*T*_inf_). This form is instructive, both because of its close relation to the definition of *R*_0_ via *β* and *γ*, and because calculating 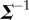 directly may be computationally infeasible for even moderately complicated models, but it can be relatively straightforward to calculate *P*_pos_ and 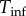 numerically (see §2 of the electronic supplementary material).

### 2.2. Dependence of R_*_ on movement rate, *R*_0_, heterogeneity and implications for control

#### 2.2.1. Features of R_*_

**Figure 1.**
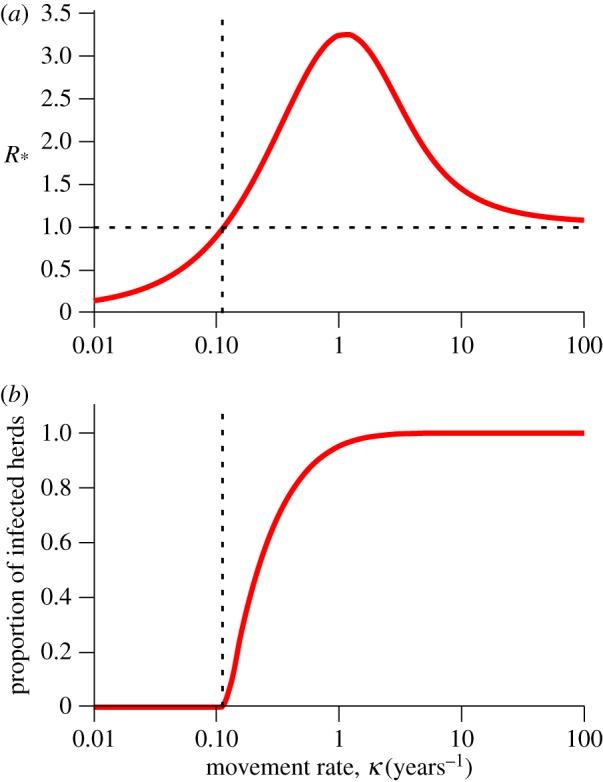
R_*_ is a threshold parameter for persistence in the metapopulation. (*a*) R_*_ versus movement rate *κ* in the metapopulation SIS model (see §1.1 of the electronic supplementary material). (*b*) The quasi-equilibrium proportion of infected herds, for the same model. The proportion of infected herds is 0 when R_*_ ≤ 1, illustrating threshold behaviour and increases to 1 as movement rates increases. Parameters are *μ* = 1/3, *γ* = 10 and *β* = 14.

In this section, we illustrate the features of R_*_ within a metapopulation of herds using SIS model dynamics. We use the formulation of *R*_0_ that reflects the primary infective's total capacity to generate secondary cases, irrespective of movement between herds (see §1.1). Then, for an underlying *R*_0_ > 1, R_*_ is zero in the absence of movements, rises above 1 as the movement rate increases, peaks at an intermediate movement rate, and then declines to 1 from above (figure 1*a*). Note that, for an underlying *R*_0_ ≤ 1, R_*_ approaches 1 for large movement rates from below (not shown). R_*_ provides a threshold for persistence of infection in the metapopulation as indicated by the quasi-equilibrium proportion of infected herds: zero for R_*_ ≤ 1, and greater than zero for R_*_ > 1 (figure 1*b*).

R_*_ initially rises, because the disease multiplies within the herd before infectives are exported to other herds via movement. However, R_*_ eventually declines as it becomes more likely that the primary infective leaves the herd before it has a chance to transmit infection within the herd (or recover or die). This results in an intermediate peak occurring when movement is low enough that the disease is sustained within the herd, but fast enough that it can reach other herds before being removed by stochastic extinction.

**Figure 2.**
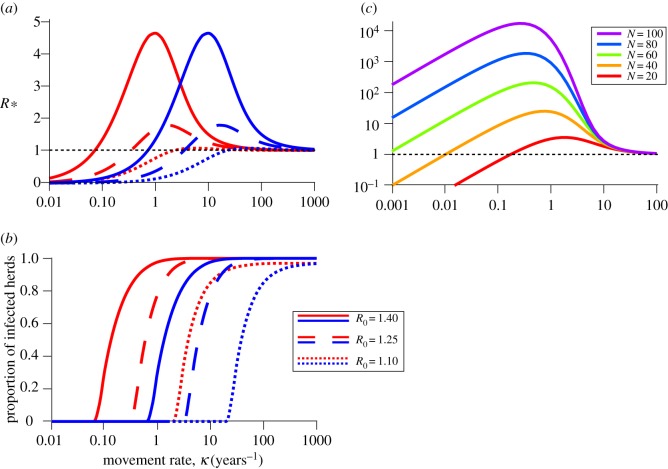
Impact of *R*_0_, herd size and movement rate on R_*_. (*a*) R_*_ in the metapopulation SIS model (see §1.1 of the electronic supplementary material). (*b*) Quasi-equilibrium proportion of infected herds in the same model, versus movement rate *κ* for slowly progressing (red) and rapidly progressing (blue) diseases, and for varying *R*_0_. The R_*_ peak occurs for lower movement rate in the slowly progressing disease, and increases rapidly with *R*_0_. Parameters are *μ* = 1/3, *γ* = 10 and 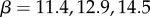. (*c*) R_*_ (log scale) versus increasing herd size *N* in the same model. The R_*_ peak increases roughly exponentially with *N*. Parameters are *μ* = 1/3, *γ* = 10 and *β* = 17.5.

The peak in R_*_ increases in magnitude and shifts to lower movement rates as *R*_0_ increases (figure 2*a*) with a corresponding shift in the threshold for persistence (figure 2*b*). In addition, for the same *R*_0_, slowly progressing diseases (i.e. those with a low recovery rate; figure 2*a*, red curves) have a higher equilibrium proportion of infected herds at the same movement rate than a rapidly progressing disease (i.e. those with a high recovery rate; figure 2*a*, blue curves), with corresponding shifts in the threshold for persistence (figure 2*b*).

From the expression for R_*_ (equation (2.1)), we see that herd size *N* contributes to R_*_ via the number of movements out of the herd and also via its potential effect on the prevalence when infected, *P*_pos_, and the persistence time, *T*_inf_. The net result is substantial nonlinear increases in R_*_ with increased herd size, but relatively little change in the position of the peak in R_*_ (figure 2*c*).

#### 2.2.2. Implications for control

**Figure 3.**
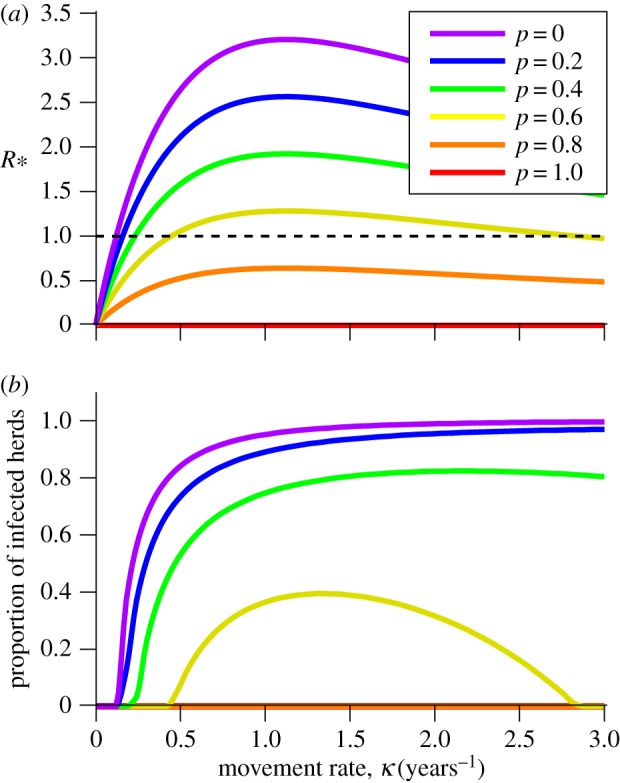
Effect of movement-based controls on R_*_. (*a*) R_*_ in the metapopulation SIS model (see §1.1 of the electronic supplementary material) and (*b*) equilibrium proportion of infected herds, in the same model, plotted against movement rate *κ*, and for increasing disease prevention *p*, from *p* = 0 (violet) to *p* = 1 (red), shown at intervals of 0.2. Effective 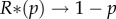 for large movement rates and the disease can persist whenever effective R_*_(*p*) > 1. This creates an intermediate range of movement rates for which the disease is able to persist (‘islands’ of persistence) at the specified level of control. Parameters are *μ* = 1/3, *γ* = 10 and *β* = 14.

To simulate the effect of control measures, we define, for any set of individuals selected to move, *p* to be the proportion of infectious individuals that are treated or prevented from bringing the disease into another group. This interception occurs at the point of movement, therefore, only the remaining proportion 1 − *p* of individuals successfully carry the infection to another herd. We therefore obtain the effective reproduction ratio in the presence of control, R_*_(*p*), which is
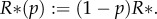
This leads to an important result. Disease can spread in the presence of control only if R_*_(*p*) remains above 1, leading to ‘islands’ of persistence (figure 3*b*).

Using our metapopulation model (see §2 of the electronic supplementary material), we calculated R_*_(*p*) for a range of levels of disease intervention and the corresponding equilibrium proportion of infected herds in the metapopulation. Near the R_*_ peak (intermediate movement rate), even high levels of disease intervention may fail to control the disease (figure 3*a*, yellow curve), but when the movement rate is high even low levels of control may be sufficient to reduce R_*_(*p*) to below 1 and prevent the disease from spreading.

Consequently, for a range of intermediate values of the movement rate the infection persists in the meta-population for a given level of control, but at higher or lower movement rates, infection cannot persist under the same level of control (e.g. figure 3*b*, yellow curve). The range of values of the movement rate for which disease persists depends on the level of control applied.

## 3. Next-generation matrix approach for heterogeneous systems

In this section, we demonstrate how R_*_ may be constructed for the more complex disease systems and explore the impact of such heterogeneity on R_*_.

### 3.1. Multiple disease categories

Consider a disease with two possible infectious states: types *A* and *B* (e.g. a regular shedder and a supershedder). As above, we assume the herd size *N* is constant, so the herd has potential disease states *x_a_*_,*b*_, where *a* and *b* correspond to the number of individuals in a herd in categories *A* and *B*, respectively; here 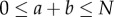, and the disease-free state is 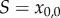. The state space ***x***(*t*) is obtained by enumerating over all the possible infected herd states, and then the NGM with large domain ***K***_L_ needed to calculate R_*_ may be constructed by proceeding as before. Here, we illustrate the process.

Because infection in a herd is initiated by one individual, a disease with a single infectious category has one entry point *x*_1_, whereas with two infectious categories, there are two entry points: *x*_1,0_ and *x*_0,1_, depending on which type of infective first enters the disease-free herd. Therefore, in this case, the transmission matrix ***T*** (and hence also ***K***_L_) has two rows with non-zero entries, and therefore, ***K*** is a 2 × 2 matrix.

Any given herd state can be reached by a limited number of adjacent states via the various event types, and each row in 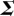 will have as many entries as possible transitions (e.g. here under the constraint of fixed herd size *N* there are four, corresponding to increases in *A* and *B* due to infection, and decreases owing to recovery or mortality). The matrix 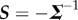 of sojourn times is dense, but as above we exploit the fact that the columns corresponding to the entry points determine the total infection duration *T*_inf_, which now depends on which entry point is reached (i.e. we must now consider both 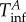 and 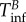).

Extracting the elements of ***K***_L_ relating to the entry points, we obtain the reduced NGM ***K***, which has entries:
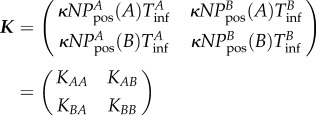
where, for example, 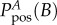 means the expected prevalence of *B* given entry point *A*, and 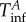 is the expected duration of the infection in the herd given entry point *A*.

Here, *K_BA_* is the number of secondary herds initially infected by a class *B* individual that are caused by a primary infected herd initially infected by a class *A* individual. This is convenient, as it means that the entries in ***K*** may be computed via simulation of a single herd, by seeding an infection with an infective of category *j*, and approximating each entry *K_ij_* as the number of category *i* infectives moving to susceptible herds (averaged over a sufficiently large number of repeated simulations).

Note that because R_*_ is a function of all entries in ***K***, it is possible that a disease with multiple infectious categories may have multiple R_*_ peaks (this phenomenon is just distinguishable in the curve for BHV in figure 5).

#### 3.1.1. Example illustrating the impact of within-herd heterogeneity in infectiousness

Consider a livestock infection such as *E. coli* O157, which exhibits substantial heterogeneity between individuals in transmissibility [37–39]. Here, we characterize this heterogeneity using a simple low shedder–high shedder version of an SIS model which we call the SLHS model (see §1.2 of the electronic supplementary material). We assumed that susceptibles *S* become either supershedders *H* (high) or regular infectives *L* (low), with probabilities *p* and 1 − *p*, respectively, and that supershedders are *η* times more infectious than regular infectives. To illustrate the effect of heterogeneity on R_*_, we chose *η*, *p* and a normalizing constant (see §1.2 of the electronic supplementary material) to ensure that *R*_0_ remains constant as we vary the relative contributions to transmission from the low and high shedders.

We calculate R_*_ by simulating herds where the initial infection is either a low or high shedder, and each case populates a column in the NGM
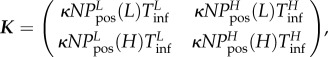
as described in §3.1.

**Figure 4.**
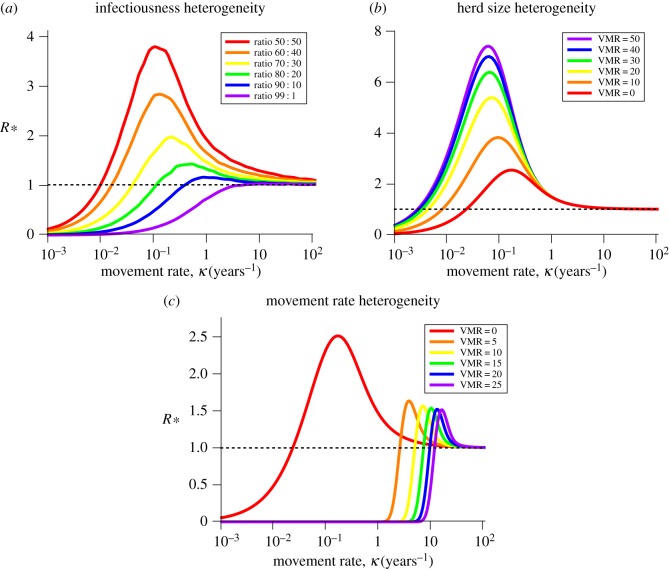
Impact of heterogeneity on R_*_. R_*_ versus movement rate *κ* with (*a*) increasing heterogeneity between low and high shedders. Parameters are as the *E. coli* O157 model (see §1.2 of the electronic supplementary material); (*b*) increasing heterogeneity in herd size *N* in the SIS model (see §1.1 of the electronic supplementary material) and (*c*) increasing heterogeneity in movement rate *κ* in the same model. Parameters are *μ* = 1/3, *γ* = 1, *β* = 1.75. The homogeneous case in each plot is shown in red, moving to purple with increasing heterogeneity. R_*_ is maximized by homogeneous infectiousness and movement, but maximized by heterogeneous herd size, as larger herds contribute disproportionately more to transmission.

The highest R_*_ comes from the most homogeneous disease transmission (figure 4*a*). The explanations for this are a combination of (i) susceptible depletion, i.e. while *R*_0_ (which ignores susceptible depletion) remains constant, the initial supershedder, when highly infectious, is unable to reach its full potential owing to a lack of susceptibles and (ii) an increased chance of stochastic extinction when the majority of the transmission is due to the relatively rare supershedders.

### 3.2. Between-herd heterogeneity

We now consider the case of an SIS disease with heterogeneity in herd size *N* and movement rate *κ*. Suppose the population consists of *n* herd types where a proportion *p_j_* of herds have *N_j_* individuals and *per capita* movement rate *κ_j_*. The mean herd size is 
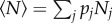
, and the mean movement rate is 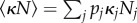. Here the state vector is of size 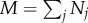, representing the numbers of infectives for each herd size 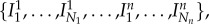 where 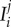 is the number of herds of type *j* with *i* infectives.

The transmission matrix ***T*** is of size *M* × *M*, but has only *n* entry points, corresponding to 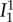 to 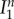 and is therefore composed entirely of zeros except for *n* rows. The transition matrix 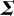 is an *M* × *M* block matrix, where each diagonal block is a tridiagonal submatrix of size *N_j_* × *N_j_* (because the only state change is to increase or decrease the number of infectives by 1), and each off-diagonal block is an *N_i_* × *N_j_* zero submatrix (because herds do not change size). 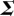 is tridiagonal, and so 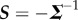 has dense diagonal blocks. Consequently, ***K***_L_ is size *M* × *M*, and ***K*** is *n* × *n*.

As above, we avoid calculation of ***T*** and 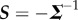 and proceed by direct calculation of the elements of ***K***. Because herd ‘susceptibility’ and ‘transmissibility’ are independent of who is infecting whom, each entry *K_ij_* only requires computation of the expected persistence time and the expected prevalence when infected for each herd type *j* denoted by 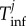 and 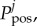 respectively. Then, each *K_ij_* is the number of secondary infections in a herd type *i* corresponding to entry from a herd of type *j*. Because
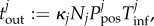
infectives leave herds of type *j*, and enter disease-free herds of type *i* with probability
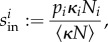
this gives
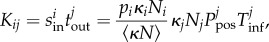
and so
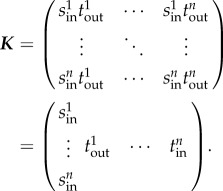


Because ***K*** is the outer product of two vectors, and so all rows of ***K*** are linear multiples of each other, there is just one non-zero eigenvalue, given by the sum of the diagonal elements of ***K***, i.e.3.1
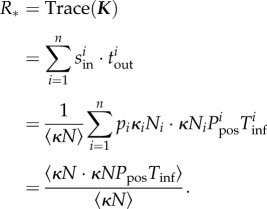
This form has natural parallels with the expression for *R*_0_ on a random network:
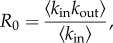
where *k*_in_ and *k*_out_ refer to the number of infectious in and out links per node [40]. In our expression, the number of outward infectious links also captures the within-node disease dynamics via the terms 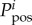 and 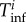 for the expected on farm prevalence while infected and the expected duration of infection.

Note that to maintain herd sizes, we assume that movement in *κ*_in_ equals movement out *κ*_out_. However, in more complex scenarios, such as asymmetric cattle movement, this restriction may be relaxed, relying on within herd dynamics to maintain herd size. This would lead to more complex expressions for 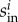 and 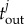, however this is beyond the scope of this paper.

### 3.3. Heterogeneity in herd size *N* and movement rate *κ*

Now using the NGM method described in §3.2, we examine how R_*_ in the SIS model depends on heterogeneity in herd size *N* and movement rate *κ*. Heterogeneity is created by keeping a fixed mean, but varying the variance-to-mean ratio of a gamma distribution (discretized in the case of herd size).

R_*_ is higher in populations with greater heterogeneity in herd size (figure 4*b*), but lower in populations with greater heterogeneity in movement rate (figure 4*c*), which can be explained heuristically as follows. Larger herds are associated with a lower chance of stochastic extinction [41] and therefore, a larger *T*_inf_. Thus, larger herds will have a greater *N* and *T*_inf_ and therefore contribution disproportionately to R_*_.

If each herd has its own *per capita* movement rate *κ_i_*, then each herd will contribute differently to R_*_. As there is a movement rate κ* that maximizes R_*_, the highest R_*_ should occur in the homogeneous case where *κ_i_* = κ* for all herds. Any heterogeneity in *κ_i_* should reduce R_*_, as some herds will contribute less to R_*_. Consider the extreme case, where the population is composed of two groups, a small number of herds with high *κ_i_*, which contribute R_*_ ≈ 1, and a large number of herds with low *κ_i_*, which contribute R_*_ ≈ 0. Therefore, R_*_ is maximized by homogeneous movement, and this result is indeed shown in figure 4*c*.

## 4. R_*_ in four important livestock disease systems

We consider four important and epidemiologically different cattle diseases: BVDV, BHV, *Mycobacterium avium* ssp *paratuberculosis* (ParaTB, the pathogen responsible for Johne's disease), and *Escherichia coli* O157 (*E. coli* O157). Models and parameters for the first three are based on non-spatial deterministic models described by Carslake [42], whereas those for *E. coli* O157 are based on [37,43], (see §1 of the electronic supplementary material).

We calculated R_*_ for each model by populating the NGM ***K*** directly, obtaining each entry *K_YX_* by simulation, introducing a single individual of infectious type *X* to a susceptible herd, and counting the number of infectious type *Y* leaving the herd via movement until the infection died out in the primary herd. To find the associated quasi-equilibrium proportion of infected herds, we also simulated a metapopulation of *n* = 100 herds each with *N* = 50 individuals. Our assumption of a homogeneous metapopulation means that we assume undirected movement between herds, and that movements are equally likely between any herds.

We considered movement rates *κ* between 0.0001 and 100 per year (see §2 of the electronic supplementary material for details on the methods used). Cattle typically move around one to four times during their lifetime, which has a mean of around 3 years [44]. Consequently, the range of movements of most interest is around *κ* = 1 (one movement per animal per year). However, these models are intended to expose the range of behaviours of R_*_, rather than make precise predictions, and thus we consider a wider range of movement rates than is typically recorded.

**Figure 5.**
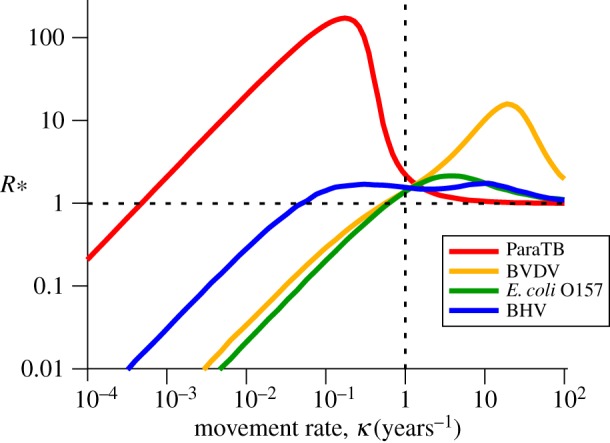
R_*_ versus movement rate *κ* for four different cattle diseases: BVDV, BHV, ParaTB, and *E. coli* O157 (see §1 of the electronic supplementary material for full details). Around typical cattle movement rates of *κ* = 1, all diseases here have R_*_ > 1, and hence are able to spread between herds, however R_*_ is maximized for higher *κ* in BVDV, and lower *κ* in ParaTB. Owing to long persistence times of infection, some simulations for ParaTB were truncated, and so the value of R_*_ presented is actually a lower bound on the true value.

For each of the exemplar diseases, R_*_ reaches an intermediate peak above 1 for some intermediate movement rate (figure 5). The slowly progressing ParaTB has a peak at low movement rates, whereas the rapidly progressing BVDV has a peak at high movement. BHV and *E. coli* O157 have intermediate transmission rates, and thus peak at intermediate movements; however, the two categories of infective for BHV lead to a double peak.

**Figure 6.**
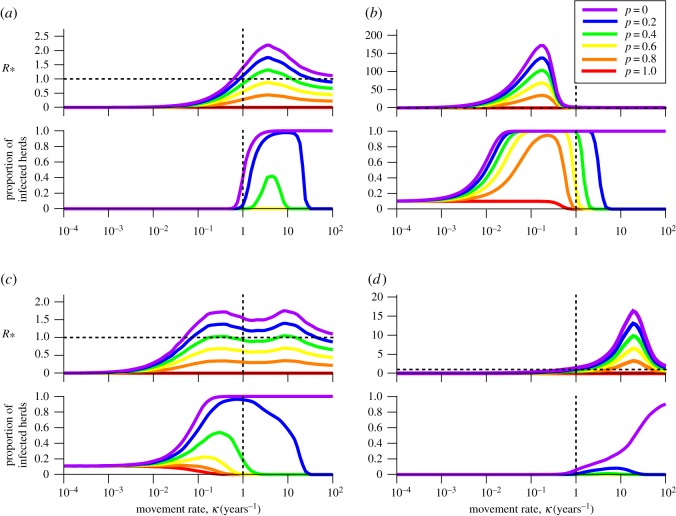
R_*_ and proportion of infected herds against movement rate *κ* for (*a*) *E. coli* O157, (*b*) ParaTB, (*c*), BHV and (*d*) BVDV. *n* = 100 herds were simulated (see §1 of the electronic supplementary material for full details). While not intended as an exact representation of reality, the vertical dashed line at *κ* = 1 represents the area roughly closest to real life movement rates. Higher *κ* would make *E. coli* O157 and BVDV more persistent, while lower *κ* would favour ParaTB. The highest R_*_ is seen in ParaTB (*T*_inf_ is extremely high for low *κ*, and the value give for R_*_ here is only a lower bound), and this corresponds to ParaTB being difficult to treat when *κ* is low. Note the double peak for R_*_ in BHV (*c*), and the green line dips below 1 around *κ* = 1; while the proportion of infected herds is calculated at *t* = 20, the disease may ultimately be unable to persist for longer time periods.

Comparing R_*_ with the proportion of infected herds (figure 6) shows that while all diseases achieve the maximum proportion of infected herds for high movement rates in the absence of disease intervention, even a relatively weak control effort (*p* = 0.2, indicating that 20% of infected individuals are identified and treated, blue lines) is sufficient to control the disease at high movement rates. With low movement rates, ParaTB is difficult to control (even a high control effort fails to control infection), but with greater movement effective control becomes easier.

## 5. Discussion

The work reported here is motivated by the desire to control disease in regional and national livestock populations and addresses the lack of suitable metrics for determining the level of effort required when movement-based disease control is used to reduce disease transmission that is primarily driven by movement (trading) of livestock.

We describe a novel formulation of the threshold for disease spread in a structured population, R_*_, that explicitly captures group to group transmission via animal movements. While a number of previous studies have addressed the impact of group structure on disease invasion, some analytically [29,30,45] and some via statistical and simulation methods [19,24], this is the first demonstration of a threshold parameter for disease invasion in a metapopulation that captures within-group stochastic dynamics coupled with the explicit movement of infected individuals between groups.

Following Diekmann and Heesterbeek, we use an NGM approach to calculate R_*_ and show how this may be used for disease systems with heterogeneities and multiple infectious states. We show for a simple disease system that R_*_ is given by the intuitive expression 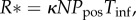 where *κ* is the movement rate, *N* is the herd size, *T*_inf_ is the expected persistence time and *P*_pos_ is the expected prevalence in an infected herd. Note that this factorization of R_*_ is non-trivial and accounts for the fact that prevalence and persistence time may be correlated. Pellis *et al.* [46] make a similar observation about their factorization of R_*_ for household models.

A key feature is the presence of a peak in R_*_ at intermediate movement rates. This novel observation arises, because we have explicitly modelled the herds' gain and loss in infectives that occurs when disease is spread by livestock movements. In household models where the contact process resulting in disease transmission is captured phenomenologically, there would be no such peak [30,32].

The R_*_ peak depends on the interaction between movement rate *κ*, the within-herd disease persistence time *T*_inf_, and the expected prevalence *P*_pos_ in an infected herd. Movement contributes directly to R_*_, but crucially also removes infectives from the herd, and therefore can reduce *T*_inf_ and *P*_pos_. It is this trade-off that leads to the characteristic intermediate peak. Theoretically, for very high movement rates, an infective animal would arrive on farm and then immediately leave with virtually no opportunity to make infectious contacts, recover or die; for this reason R_*_ tends to 1 at high movement rates.

The peak in R_*_ has important consequences for control directed at livestock moving between herds. The degree of control effort required also peaks at intermediate movement rates, and consequently a given level of control may be sufficient to prevent persistence at low or high movement rates, but be insufficient over a range of intermediate movement rates. This phenomenon arises, because increased movement exposes more animals to testing, with the consequence that controls need to be less effective at identifying infected animals at high movement rates to achieve a given reduction in prevalence.

R_*_ increases dramatically with increased herd sizes that substantially increase the persistence of infection. In addition, rather modest values of *R*_0_ can, depending on the disease system, be associated with values of R_*_ that are orders of magnitude larger. This finding indicates that for some disease systems control directed at reducing *R*_0_ may be more effective than controls directed at animals moving between holdings.

We also demonstrated that R_*_ is maximized when there is the least heterogeneity between farms in movement rates and when there is the least individual variation in infectiousness; conversely, increasing heterogeneity in herd size increases R_*_.

Our exemplar disease models and their parametrizations were selected, not to give precise predictions, but to provide a range of R_*_ behaviours across four important livestock diseases. The different disease dynamics result in quite different R_*_ profiles, leading to potential trade-offs between the control of different diseases. All the exemplar diseases have low R_*_ near the intermediate *per capita* movement rate of one movement per year, but our predictions indicate that ParaTB and BVDV would have much higher R_*_ at lower movements for ParaTB and at higher movements for BVDV. ParaTB (Johne's disease), a slowly progressing disease which persists in a herd for a long time, has an R_*_ that peaks at low movement rates indicating that it might prove difficult to control if movement rates were reduced; however, increasing movement rates slightly could expose it to sufficient intervention that it would be unable to spread between herds.

In contrast, *E. coli* O157, a rapidly progressing disease with an R_*_ peak at higher movement rates may be better able to persist in the face of movement-based controls at higher movement rates. BHV, which can also persist in herds for long periods is able to invade at lower movement rates than would be needed for invasion by *E. coli* O157 or BVDV. These findings concur with the observations that chronic diseases are more likely to invade than acute diseases with the same *R*_0_ [29].

The consequence of the differing R_*_ profiles is that if, for example, movement restrictions were put in place to reduce *E. coli* O157, ParaTB could become more difficult to control via movement-based controls. On the other hand, if movement rates increased, ParaTB could be easier to control via movement-based controls, at the cost of increased prevalence of *E. coli* O157. Overall, our results indicate that at current livestock movement rates, disease control implemented at the point of between-farm movement alone can be sufficient to control some pathogens, but for infections such as ParaTB control at herd level is likely to be needed in addition.

Inevitably, the models analysed in this paper include a number of simplifying assumptions; nevertheless, our methodology (a key result of this paper) is applicable to more realistic scenarios. The extensive explorations presented in this paper indicate that the following results will hold in more complex scenarios: R_*_ will peak and decline, leading to ‘islands’ of persistence when control is implemented. In addition, we anticipate that different diseases will have different R_*_ profiles, potentially leading to conflicting requirements when controlling multiple diseases.

In summary, our formulation of R_*_ provides novel theoretical insights into the likely effectiveness of alternative control strategies and an important addition to the selection of tools available to epidemiologists to be used in conjunction with *R*_0_ for disease control in livestock systems.

## Supplementary Material

Supporting information
