# Supplementary material for: Complex responses to movement-based disease control: when livestock trading helps
Source: J R Soc Interface. 2017 Jan;14(126):20160531. doi: 10.1098/rsif.2016.0531 (PMC5310727; doi:10.1098/rsif.2016.0531)
Supplement: Supporting information [file rsif20160531supp1.pdf]

Supporting Information

Jamie C. Prentice[1,2*], Glenn Marion[3], Michael R. Hutchings[4], Tom N. McNeilly[5], and Louise Matthews[1,2]

[1]Institute of Biodiversity, Animal Health and Comparative Medicine, College of Medical, Veterinary and Life Sciences, Glasgow, G61 1QH
[2]Boyd Orr Centre for Population and Ecosystem Health, University of Glasgow
[3]Biomathematics and Statistics Scotland, Edinburgh, EH9 3JZ
[4]Disease Systems, SRUC, Edinburgh, EH9 3JG
[5]Moredun Research Institute, Penicuik, EH26 0PZ
[*]jamie.prentice@glasgow.ac.uk

30th November 2016

1 Specific disease models

For bovine herpes virus, bovine viral diarrhoea virus, and ParaTB, we used stochastic analogues of the deterministic metapopulation models used in Carslake *et al.* [1].

Since Carslake *et al.* do not clearly specify demography, we implemented it as follows: mortality occurs at *per capita* rate μ, recruitment (birth or buying new animals to replace stock) at rate μN, where N is the herd size. Mortality and recruitment were coupled, to ensure that the herd size remains constant. Where vertical transmission was a component of a model, recruited individuals were introduced into an infected class with the appropriate probability.

Movements were assumed to occur at *per capita* rate κ. For the purposes of calculating R_*, infectives moving out of a herd were immediately replaced by a new individual in order to maintain the herd size at N; the status of replacement individual (e.g. susceptible or infected) was given by proportion of each type of individual amongst all other herds in the metapopulation.

In each case, the population was seeded with one initial infective, and allowed to run until the herd was free from infectives. This was repeated for each category of infective, in order to populate the NGM $\boldsymbol{K}$.

1.1 General SIS model

The stochastic event table for the SIS model with the given dynamics is as follows:

Change	Events	Rate
$S \to I$	Infection	$\beta SI/N$
	Movement	$\kappa P_I S$
$I \to S$	Birth & Death	μI
	Recovery	γI
	Movement	$\kappa P_S I$

Note that we exclude events which have no effect on the state, e.g. all deaths are coupled to replacement by new susceptibles, so the state change for mortality of S is $S \to S$. In later models, we do not specify every single movement combination, and instead just note the general case $X \to Y$ at rate $\kappa P_Y X$.

In all cases where the SIS model was used, mortality $\mu = 1/3$ [2], while values used for β and γ are specified in the relevant figures.

1.2 Escherichia coli O157

We used an SLHS model, where susceptibles S become either super shedders H (high), or regular shedders L (low), with probabilities p and $1 - p$ respectively. Super shedders are η times more infectious than regular infectives.

$$\begin{aligned}
\dot{S} &= \mu(N - S) - c\beta(L + \eta H)S/N + \gamma(L + H)\\
\dot{L} &= -(\mu + \gamma)L + (1 - p)c\beta(L + \eta H)S/N\\
\dot{H} &= -(\mu + \gamma)H + pc\beta(L + \eta H)S/N
\end{aligned}$$

where

$$N = S + L + H$$

and where the parameters are as specified in Table 1. We took $R_0 = 1.5$ [3], and assumed a mean infectious period of 4 weeks. The stochastic event table is shown in Table 2.

We calculate R_* for this model numerically in Section 3.1 of the main manuscript using the simulation approach discussed in Section 2, but we also calculate R_0 using the NGM method, to show that R_0 does not depend on the probability of super shedding p.

Using the NGM method, the Jacobian matrix of the linearised subsystem is

$$\boldsymbol{A} = \begin{pmatrix} -\mu - \gamma + (1-p)c\beta & +(1-p)c\beta\eta \\ +pc\beta & -\mu - \gamma + pc\beta\eta \end{pmatrix}$$

which decomposes into the transmission matrix

$$\boldsymbol{T} = \begin{pmatrix} (1-p)c\beta & (1-p)c\beta\eta \\ pc\beta & pc\beta\eta \end{pmatrix} = c\beta \begin{pmatrix} 1-p \\ p \end{pmatrix} \begin{pmatrix} 1 & \eta \end{pmatrix}$$

and the transition matrix

$$\boldsymbol{\Sigma} = \begin{pmatrix} -\mu-\gamma & 0 \\ 0 & -\mu-\gamma \end{pmatrix} = -(\mu+\gamma) \begin{pmatrix} 1 & 0 \\ 0 & 1 \end{pmatrix}$$

and so the NGM is

$$\boldsymbol{K} = -\boldsymbol{T}\boldsymbol{\Sigma}^{-1} = \frac{c\beta}{\mu+\gamma} \begin{pmatrix} 1-p \\ p \end{pmatrix} \begin{pmatrix} 1 & \eta \end{pmatrix}$$

which gives

$$R_0 = \frac{c\beta}{\mu+\gamma}(1-p+\eta p)$$

Therefore we choose $\eta = (1/p-1)^2$ to ensure the correct transmissibility, and $c = 1/(p-1)$ to normalise transmission, and then $R_0 = \beta/(\mu+\gamma)$ is independent of p.

1.3 Bovine herpes virus (BHV)

We used an SILI model, where susceptibles S become infectives I, then recover to a latent state L, from which they may later relapse to I again.

$$\begin{aligned} \dot{S} &= +\mu(N-S) - \beta SI/N \\ \dot{I} &= -\mu I + \beta SI/N - \gamma I + \alpha L \\ \dot{L} &= -\mu L + \gamma I - \alpha L \end{aligned}$$

where

$$N = S + I + L$$

and where the parameters are as specified in Table 1. The stochastic event table is shown in Table 2.

1.4 Bovine viral diarrhoea virus (BVDV)

We used an SIPR model, where individuals start as susceptibles S, then become infectives I, and recover to resistant R. Newly born individuals can become persistent shedders P with vertical transmission probability p_{v}.

$$\begin{aligned} \dot{S} &= +(1-p_{\mathrm{v}})\mu(N-S) - \beta S(I+P)/N + \gamma_1 P \\ \dot{I} &= -\mu I + \beta S(I+P)/N - \gamma_2 I \\ \dot{P} &= +p_{\mathrm{v}}\mu(N-S) - \mu P - \gamma_1 P \\ \dot{R} &= -\mu R + \gamma_2 I \end{aligned}$$

where

$$\begin{aligned} N &= S + I + P + R \\ p_{\mathrm{v}} &= (p_1 P + p_2 I)/N \end{aligned}$$

and where the parameters are as specified in Table 1. The stochastic event table is shown in Table 2.

1.5 *Mycobacterium avium* ssp *paratuberculosis*

This is the pathogen responsible for Johne's disease, and is also known as Map or ParaTB. We used an SLHC model, where susceptibles S become low shedders L, then progress to high shedders H, and finally progress to clinically infected C. Newly born individuals become low shedders L with vertical transmission probability p_{v}.

$$\begin{aligned} \dot{S} &= +(1-p_{\mathrm{v}})\mu(N-S) - h - \gamma C \\ \dot{L} &= +p_{\mathrm{v}}\mu(N-S) - \mu L + h - \alpha_1 L \\ \dot{H} &= -\mu H + \alpha_1 L - \alpha_2 H \\ \dot{C} &= -\mu C + \alpha_2 H - \gamma C \end{aligned}$$

where

$$\begin{aligned} p_{\mathrm{v}} &= [p_1(L+H) + p_2 C]/N \\ h &= [\beta_1 L + \beta_2(H+C)]S/N \end{aligned}$$

are vertical and horizontal transmission, and where the parameters are as specified in Table 1. The stochastic event table is shown in Table 2.

2 Simulation methods

From an ease of computation perspective, there are several approaches to calculating R_*, depending on model complexity and disease dynamics. These methods may involve calculation of T_{inf} and P_{pos}, or by counting the number of infectives leaving the primary herd.

The Master Equation [4] is a set of differential equations that describe the time evolution of the probability distribution of all possible states in a stochastic model. The stochastic simulation algorithm [5] (also known as the Gillespie algorithm) provides samples which are identically distributed to the solution of the Master Equation.

The Master Equation can be used to calculate R_* exactly, by determining the elements T^j_{inf} and $P^j_{\mathrm{pos}}(i)$, the time infection persists following introduction of an individual of infectious class j, and the average prevalence of infectious class i during that period, then using these to populate the NGM $\boldsymbol{K}$ as described in Section 2 of the main manuscript. All figures for the SIS model were generated using the exact solution from the Master Equation.

However, the Master Equation rises in complexity with the number of possible states, and is not appropriate for models with multiple disease states and large herd size N. For example, with $N = 50$, the SIS model has 51 possible states, while the SLHC model for ParaTB has 22,100 possible states, with a correspondingly large Jacobian matrix, which can only be stored on most desktop computers as a sparse matrix.

For complex models it is more straightforward and biologically intuitive to use the stochastic simulation algorithm, proceeding straight to the NGM formulation and populating $\boldsymbol{K}$ directly. Each entry K_{ij} is

Model	Parameter	Symbol	Value
All	Herd size	N	50
	Mortality rate	μ	0.333
	Movement rate	κ	1*
SLHS	Horizontal transmission	β	39.5
	Recovery rate	γ	26
	Probability of super shedder	p	0.2*
	Super shedding factor	η	16*
	Normalising constant	c	0.25*
SILI	Horizontal transmission	β	36.5
	Recovery rate	γ	26.1
	Relapse rate	α	0.0913
SIPR	Horizontal transmission rate	β	91.3
	Vertical transmission probability (P)	p_1	0.9
	Vertical transmission probability (I)	p_2	0.65
	Recovery rate (I)	γ_1	26.1
	Disease induced mortality rate (P)	γ_2	0.438
SLHC	Horizontal transmission rate (L)	β_1	0.286
	Horizontal transmission rate (H and C)	β_2	2.86
	Vertical transmission probability (L and H)	p_1	0.09
	Vertical transmission probability (C)	p_2	0.39
	Progression rate ($L \to H$)	α_1	0.332
	Progression rate ($H \to C$)	α_2	0.130
	Disease induced mortality rate (C)	γ	4.05

Table 1: List of parameters, symbols, and values used in each of the models. All rates are annual (i.e. in units of year^{-1}), other values are dimensionless. Parameters marked with * were varied in certain cases

obtained by seeding the herd with one individual corresponding to type j, and observing the number of individuals of type i leaving the herd through movement before the disease becomes locally extinct. However, a large number of realisations of the stochastic model may be required to obtain a good approximation. All Figures in Section 4 of the main manuscript and Fig. 4a were calculated using this method.

2.1 Calculating the equilibrium proportion of infected herds

For the SIS model, iterative methods were used to determine the equilibrium proportion of infected herds, using the property that at endemic equilibrium, effective $R_* = 1$ (i.e. at equilibrium, each infected herd infects on average exactly one other infected herd during its infectious lifetime).

The equilibrium proportion may therefore be calculated by proposing a value x, which is the mean prevalence of infectives within the metapopulation P_I, then increasing x if $R_* > 1$, or decreasing x if $R_* < 1$. Since $0 \leq x \leq 1$, this may be effectively done using a binary search, until effective R_* is sufficiently close to 1, and then taking the equilibrium proportion to be x/P_{pos}.

For the livestock disease models, R_* and equilibrium proportion were calculated via stochastic simulation, using a metapopulation model with $n = 100$ herds for the latter.

References

[1] Carslake D, Grant W, Green LE, Cave J, Greaves J, Keeling M, et al. Endemic cattle diseases: comparative epidemiology and governance. Philos T R Soc B. 2011;366:1975–1986. 1

[2] Vernon MC. Demographics of cattle movements in the United Kingdom. BMC Vet Res. 2011;7(31):1–16. 1

[3] Matthews L, Low JC, Gally DL, Pearce MC, Mellor DJ, Heesterbeek JAP, et al. Heterogeneous shedding of *Escherichia coli* O157 in cattle and its implications for control. P Nat Acad Sci USA. 2006a;103:547–552. 1

[4] Keeling MJ, Ross JV. On methods for studying stochastic disease dynamics. J R Soc Interface. 2008;5:171–181. 2

[5] Gillespie DT. A general method for numerically simulating the stochastic time evolution of coupled chemical reactions. J Comput Phys. 1976;22(4):403–434. 2

**Disease**	**Change**	**Events**	**Rate**
All	$X \to Y$	Movement	$\kappa P_Y X$
SLHS	$S \to L$	Infection $\to L$	$(1-p)c\beta S(L+\eta H)/N$
	$S \to H$	Infection $\to H$	$pc\beta S(L+\eta H)/N$
	$L \to S$	Mortality & Recruitment	μL
		Recovery	γL
	$H \to S$	Mortality & Recruitment	μH
		Recovery	γH
SILI	$S \to I$	Infection	$\beta SI/N$
	$I \to S$	Mortality & Recruitment	μI
	$I \to L$	Recovery	γI
	$L \to S$	Mortality & Recruitment	μL
	$L \to I$	Relapse	αL
SIPR	$S \to I$	Infection	$\beta S(I+P)/N$
	$S \to P$	Mortality & VT	$p_{\mathrm{v}}\mu S$
	$I \to S$	Mortality & Recruitment	$(1-p_{\mathrm{v}})\mu I$
	$I \to P$	Mortality & VT	$p_{\mathrm{v}}\mu I$
	$I \to R$	Recovery	$\gamma_1 I$
	$P \to S$	Mortality & Recruitment	μP
		DIM & Recruitment	$\gamma_2 P$
	$R \to S$	Mortality & Recruitment	$(1-p_{\mathrm{v}})\mu R$
	$R \to P$	Mortality & VT	$p_{\mathrm{v}}\mu R$
SLHC	$S \to L$	Infection	h
		Mortality & VT	$p_{\mathrm{v}}\mu S$
	$L \to S$	Mortality & Recruitment	$(1-p_{\mathrm{v}})\mu L$
	$L \to H$	Progression ($L \to H$)	$\alpha_1 L$
	$H \to S$	Mortality & Recruitment	$(1-p_{\mathrm{v}})\mu H$
	$H \to L$	Mortality & VT	$p_{\mathrm{v}}\mu H$
	$H \to C$	Progression ($H \to C$)	$\alpha_2 H$
	$C \to S$	Mortality & Recruitment	$(1-p_{\mathrm{v}})\mu C$
		DIM & Recruitment	$(1-p_{\mathrm{v}})\gamma C$
	$C \to L$	Mortality & VT	$p_{\mathrm{v}}\mu C$
		DIM & VT	$p_{\mathrm{v}}\gamma C$

Table 2: Stochastic event table for each model used.
